# Exploration on surgery of malignant glaucoma with prolonged absence of the anterior chamber: a retrospective consecutive case series

**DOI:** 10.1186/s12886-023-03053-6

**Published:** 2023-07-07

**Authors:** Yan Liu, Tao Wang

**Affiliations:** Department of Ophthalmology, Beijing Key Laboratory of Ophthalmology and Visual Sciences, Beijing Tongren Hospital, Beijing Tongren Eye Center, Capital Medical Universit, No.1 Dongjiaomin Lane, Dongcheng District, Beijing, 100730 China

**Keywords:** Malignant glaucoma, Surgery treatment, Shallow anterior chamber, Prognosis

## Abstract

**Objective:**

To evaluate the surgical outcomes of anterior chamber restoration in patients with malignant glaucoma and a prolonged absence of the anterior chamber.

**Methods:**

Five patients with malignant glaucoma and a long-term absence of the anterior chamber underwent a combination of anterior pars plana vitrectomy (aPPV), phacoemulsification cataract excision, intraocular lens implantation, peripheral iridotomy (PI), goniosynechialysis (GSL) (referred to aPPV + P + I + PI + GSL) at Beijing Tongren Hospital from October 2018 to June 2021. The study compared the changes in their visual acuity, intraocular pressure (IOP) and medication requirements between the pre-surgery period and their most recent follow-up visit.

**Results:**

The five patients did not report any discomfort, such as pain, tearing, swelling, etc., in their affected eyes, and maintained a stable restoration of the anterior chamber. Among the affected eyes, only one eye demonstrated improved vision during the follow-up visit, while the remaining four eyes did not show any significant improvement. One eye underwent transscleral cyclophotocoagulation as an additional procedure, while the other four eyes did not require any further surgical intervention. In all cases, the intraocular pressure (IOP) was successfully controlled below 30 mmHg. Post-surgery, four eyes still required cycloplegia treatment, and three eyes continued to rely on eye drops to manage their IOP.

**Conclusion:**

Despite minimal improvement in vision, surgical intervention successfully restored the anterior chamber in malignant glaucoma patients with a prolonged absence of anterior chamber. This restoration contributed to alleviating subjective complaints of discomfort and delaying eyeball atrophy.

## Introduction

Malignant glaucoma, a rare and serious form of glaucoma, is typically a complication of angle-closure glaucoma surgery. It can manifest at various time intervals post-surgery ranging from hours to days or even years. The name “Malignant glaucoma” is derived from the condition’s rapid and progressive nature, leading to significant impairment of visual function, that cannot be effectively treated with conventional glaucoma treatment methods. Therefore, it is crucial to treat and diagnose it early [1, 2]. Unfortunately, some patients have suffered blindness due to the lack of timely treatment of malignant glaucoma. These patients face significant challenges as their affected eyes experience severe visual loss and anterior segment inflammatory reaction, making surgery a difficult option. Moreover, there is ongoing debate regarding whether surgical intervention should be pursued to restore the anatomical structure of the anterior segment and salvage the remaining vision. Without surgical intervention, transscleral cyclophotocoagulation (TCP) is sometimes performed, but this can lead to complications such as scleral staphyloma, eyeball atrophy, and ultimately, the need for enucleation. The absence of anterior chamber in malignant glaucoma causes considerable discomfort, significantly reducing patients’ quality of life. However, there is currently limited literature available to guide the decision-making process for patients unlikely to recover their vision. This study aims to explore the outcomes of surgical treatment in patients with long-standing disease onset, severe visual impairments, and significant anterior segmental inflammation.

## Materials and methods

### Research subjects

The research subjects consisted of those with long-term absence of the anterior chamber who received consecutive surgery in the Beijing Tongren Hospital from October 2018 to June 2021. The same surgeon completed all surgeries, and the patients signed pre-surgery informed consent. The inclusion criteria were as follows: (1) diagnosed with malignant glaucoma; (2) absence of anterior chamber for more than three months.

### Surgical methods

Upon admission to the hospital, the patients were administered conventional routine antibiotic eye drops. However, for those with high IOP, a combination of local and systemic IOP-lowering medication was administered. The objective was to effectively reduce the intraocular pressure to a normal or near-normal level. Surgical treatment was conducted under general anesthesia, and prior to the surgery, a 250 ml mannitol injection was intravenously infused approximately half an hour in advance.

Surgical methods: A 3.0-mm transparent corneal tunnel incision was made at the 11:00 position, accompanied by a 1-mm transparent corneal auxiliary incision at the 2:00 position. Due to posterior synechia of the iris, preventing pupil dilation and the subsequent adhesion of the iris to the lens capsule, a viscoelastic substance was injected into the anterior chamber to create separation. A continuous curvilinear capsulorhexis with an approximate 5–6 mm diameter was performed. In cases where injecting the viscoelastic substance proved challenging due to high IOP in two eyes, a 20G scleral puncture bayonet was used at the 10:00 position, 3.5 mm away from the corneal limbus. The scleral incision was expanded using microscopic, toothed forceps leading to the outflow of liquid and vitreous bodies. Subsequently, the vitreous bodies were excised, and the liquid in the vitreous cavities was drained through multiple repetitions. Once the IOP normalized, the viscoelastic substance was re-injected into the anterior chamber again to recreate the anterior chamber formation. The lens nucleus was completely freed within the capsular bag after hydrodissection. Ultrasonic dispersion was used to fragment the lens nucleus, followed by irrigation/aspiration (I/A) extraction of the residual cortex. The viscoelastic substance was replenished, and the intraocular lens was implanted. To avoid misalignment, the position of the artificial lens was adjusted so that the lens loop corresponds to the 3 and 9 o ‘clock positions. Pupil constriction was achieved using a diluted solution of carbachol (carbachol: normal saline = 1:1) was used to contract the pupil. Capsulotomy vannas scissors were inserted through the main incision, and in conjunction with the intraocular lens manipulator in the auxiliary incision, a peripheral iridectomy of approximately 3 × 3 mm was performed at around the 5:00 position. Following prior steps, the vitreous cutter was introduced through the main incision to the anterior chamber to sever the anterior hyaloid membrane in the peripheral iris incision [3, 4]. Using a viscoelastic substance, the peripheral chamber angle was separated at 360°, and the anterior chamber was filled with a perfusion liquid during I/A. The incision’s water tightness of the incision was assessed, and any incisions with poor closure were sutured using a 10 − 0 suture. The sutures were removed after a period of half a month to one month, as appropriate.

### Follow-up visits and outcome measures

Observation indicators in follow-up visits included vision, IOP, depth of anterior chamber, use of anti-glaucoma drugs, use of cycloplegia, surgical treatment again or not, complaint of discomforts, and post-surgical complications. The criteria of successful operation were as follows: (1) stable formation of anterior chamber: central anterior chamber greater than 4CT, peripheral anterior chamber greater than 1/2CT; (2) the anterior segment of the patient was calm without congestion, corneal opacity and other symptoms, and the appearance was normal; (3) the patient had no complaints such as eye pain or photophobia.

## Results

### General conditions

**Fig. 1:**
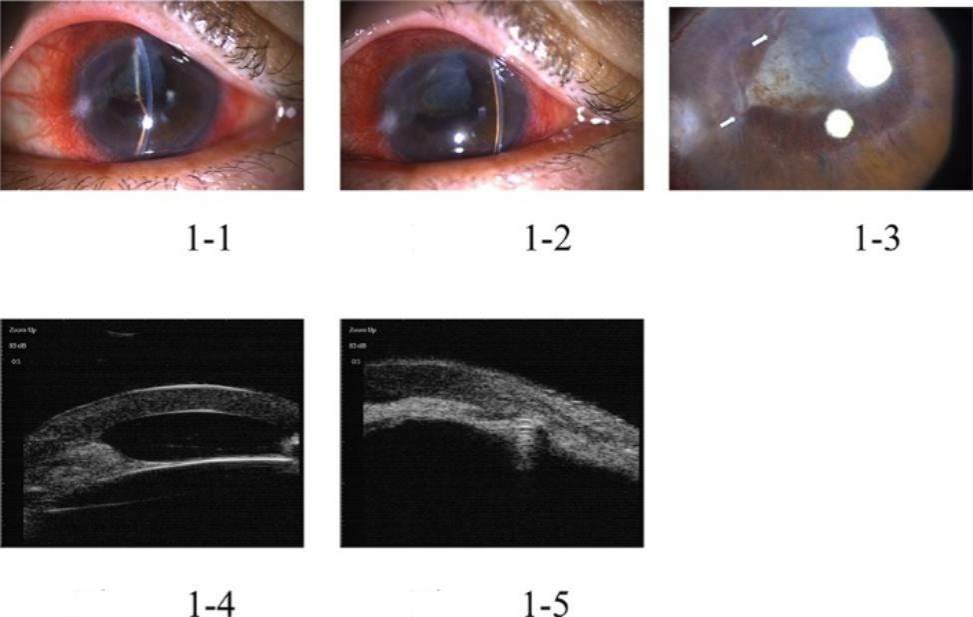
Pre-surgery pictures of anterior segment of Case 1: Pictures 1 and 2 show the disappearance of the anterior chamber, while picture 3 shows synechia of iris with anterior lens capsule (white arrow in the pictures indicated NVI). In pictures 4 and 5, preoperative UBM images show absence of anterior and posterior chamber

**Fig. 2:**
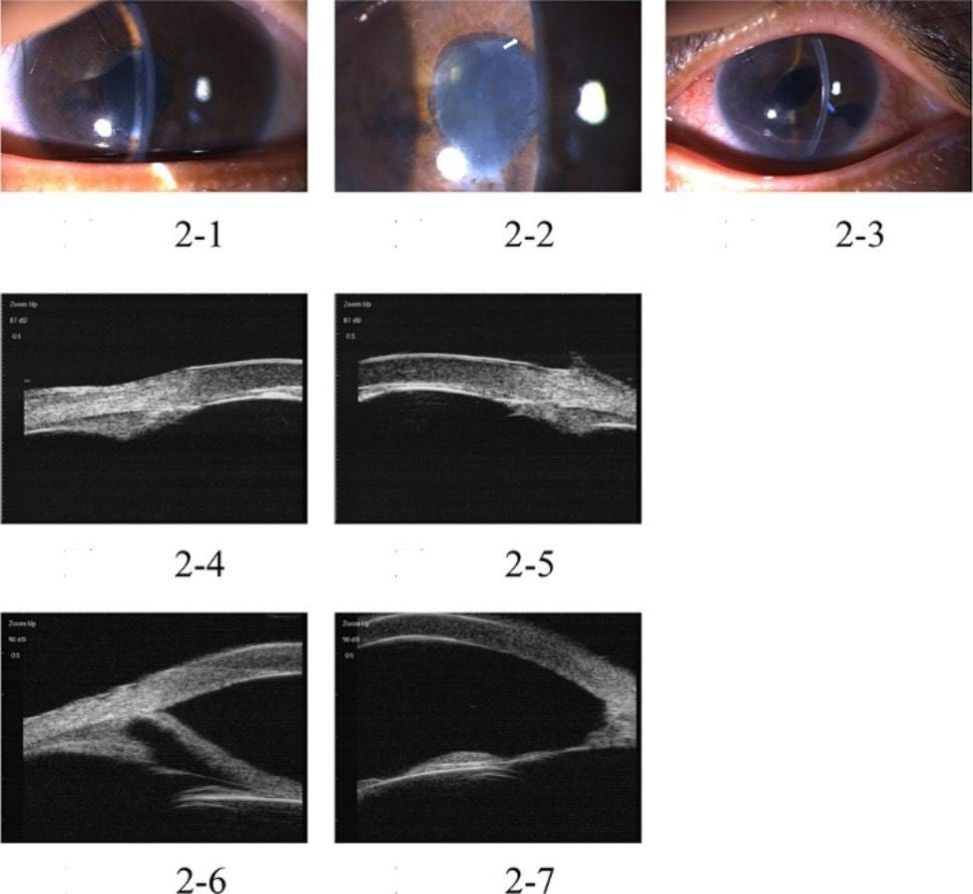
Pre-surgery and post-surgery color picture of anterior segment of Case 2, where, pictures 1 and 2 are pre-surgery color pictures, showing disappearance of anterior chamber, and the white arrow indicates CNI, picture 3 is the post-surgery color picture, showing formation of anterior chamber, and peripheral iridotomy incision in 5:00 position; In pictures 4 and 5, preoperative UBM images show that the iris is attached to the cornea and the anterior chamber is absent. UBM images six months after surgery show presence of anterior chamber and partially opening of anterior chamber angle (pictures 6 and 7)

**Fig. 3:**
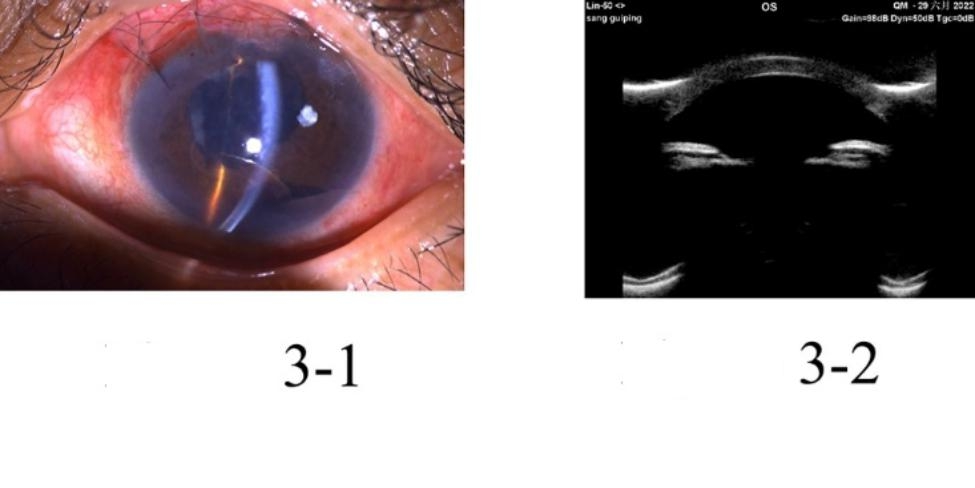
Picture 1 shows early post-surgery color picture of Case 3: mild corneal edema, formation of anterior chamber, and peripheral iridotomy incision was visible. UBM images six months after surgery shows formation of anterior chamber (Picture 2)

**Fig. 4:**
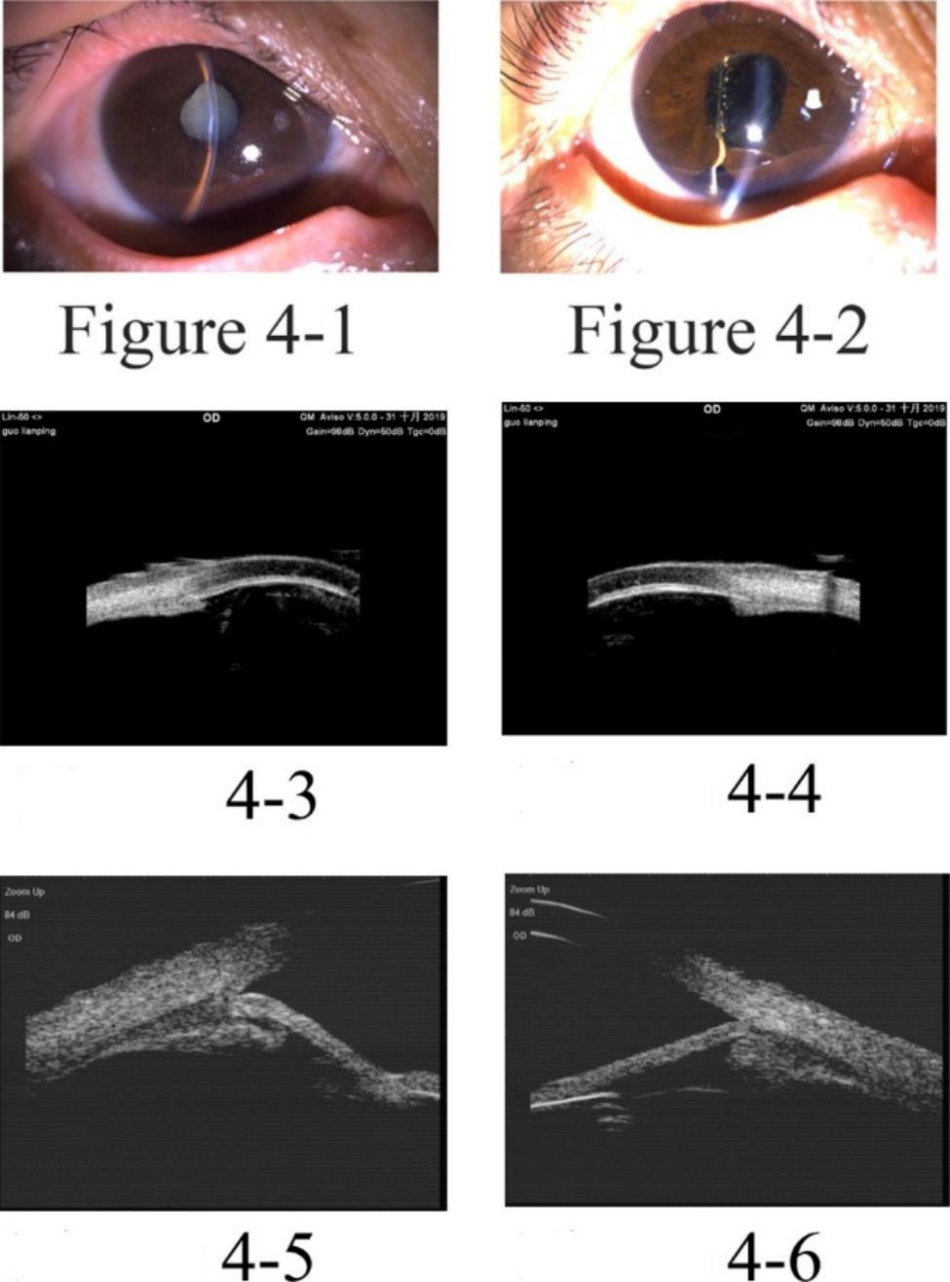
Pre-surgery and post-surgery color pictures of Case 4: (1) Disappearance of anterior chamber is visible; (2) post-surgery formation of anterior chamber is visible, and the peripheral iridotomy incision is unobstructed. In pictures 3and 4, preoperative UBM images show that the iris is attached to the cornea and the anterior chamber is absent, supporting the diagnosis of malignant glaucoma. In pictures 5 and 6, UBM images seven months after surgery show deep anterior chamber and partially opening of anterior chamber angle

**Fig. 5:**
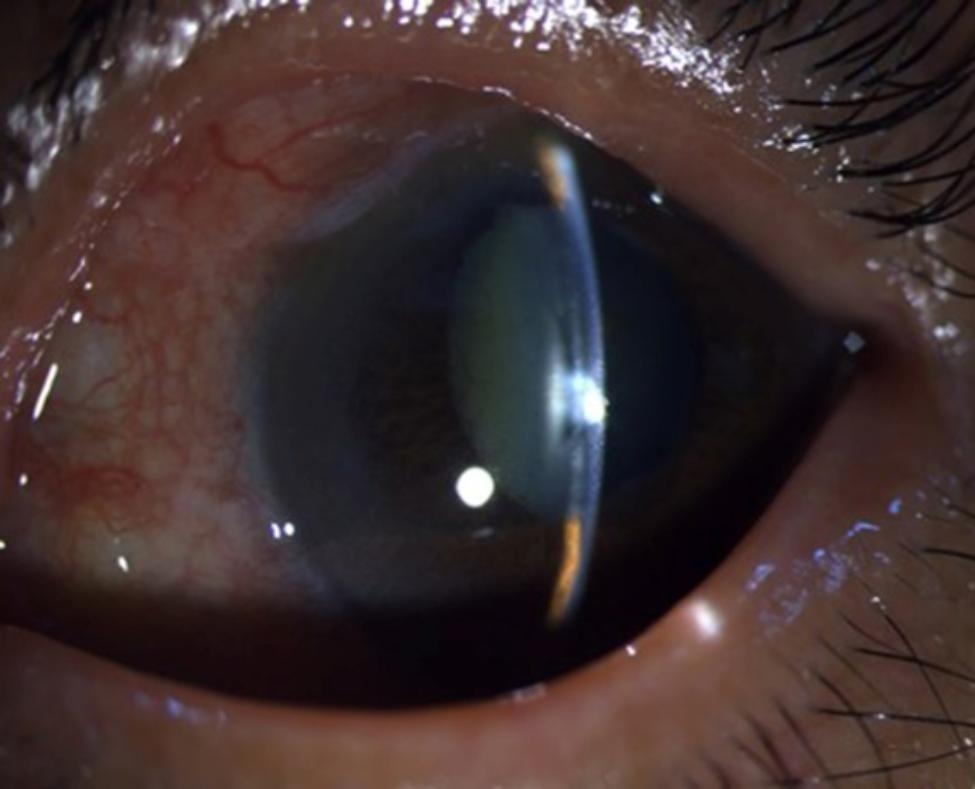
Pre-surgery color picture of Case 5: disappearance of anterior chamber is visible

The study included five patients with five affected eyes, one male and four female patients, aged 29–69 years old. Initially, all patients were diagnosed with primary angle-closed glaucoma at other hospitals and had a history of anti-glaucoma surgery. They experienced absence of their anterior chamber for a duration from 5 months to 2 years. In one eye, phacoemulsification cataract excision + intraocular lens implantation was performed due to shallow anterior chamber of malignant glaucoma after trabeculectomy. However, stable anterior chamber had not been formed in the eye. During the out-patient department in Tongren Hospital, all eyes underwent examinations using UBM and B ultrasonic scan. The examinations revealed the disappearance of the anterior chamber, but the vitreous body and retina were normal. Upon admission to the hospital, slit-lamp examinations showed that all their anterior chambers had disappeared. In one eye, the iris was attached to the corneal endothelium, and lens was also attached to corneal endothelium in one eye, resulting in anterior and posterior synechia of the iris due to serious anterior segment inflammatory reactions. Two eyes exhibited iris neovascularization, which was caused by long-term high intraocular pressure leading to anterior segment ischemia and chronic inflammatory stimulation. Regarding patients’ vision before surgery, four patients could see hand movement in front of their eyes, while one patient could count fingers in front of her eye. Regarding the pre-surgery intraocular pressure (IOP), three patients had well-controlled IOP within normal range (< 21 mmHg.), while the other two had elevated IOP, despite receiving local and systemic IOP-lowering medications. All five affected eyes received cycloplegia treatment, but still failed to form anterior chambers. One eye was implanted with an intraocular lens, while the remaining four had complicated cataracts. The patients received surgical treatment upon admission to the hospital. One patient underwent anterior pars plana vitrectomy (aPPV) + peripheral iridotomy (PI) + goniosynechialysis (GSL). The other four patients underwent phacoemulsification cataract excision + aPPV + PI + GSL. Among the latter group, three eyes were implanted with an intraocular lens, and one eye did not receive the implant (Table 1). All five eyes successfully achieved stable formation of the anterior chamber (Figs. 1, 2, 3, 4 and 5). None of the five patients reported eye discomforts such as redness, swelling, pain, photophobia, or tearing during the follow-up visits.

**Table 1:** General case and list of eye conditions

Patient No.	Gender	Age	Previous surgery	Duration without anterior chamber (m)	Iris neovascularization	Existence of lens or not	Axial length (mm)	Follow-up visit time (m)	Surgical method	Pre-surgery vision	Post-surgery vision	Pre-surgery IOP (mmHg)	Post-surgery IOP	Pre-surgery medication	Post-surgery medication	Surgery after the surgery again?
Case 1	Male	61	1, 2	5	+	No	22.22	8	aPPV + GSL + PI	HM	HM	16	11	No	No	No
Case 2	Female	29	1	12	+	Yes	21.14	10	P + I + aPPV + GSL + PI	FC	HM	18	30	A + 2	A + 2	Yes
Case 3	Female	54	1	24	-	Yes	20.47	26	P + aPPV + GSL + PI	FC	FC	52	21	A + 4	A + 3	No
Case 4	Female	66	1	8	-	Yes	22.25	27	P + I + aPPV + GSL + PI	FC	0.2	14	16	A	A	No
Case 5	Female	69	1	12	-	Yes	22.55	40	P + I + aPPV + GSL + PI	FC	FC	46	19	A + 4	A + 3	No

Note: *Previous surgeries: 1: Trabeculectomy; 2: phacoemulsification cataract excision + intraocular lens implantation (P + I);

*Pre-surgery and post-surgery medication: A: Atropine eye ointment; 1, 2, 3, 4: number of IOP-lowering eye drops used;

*Surgical method refers to the surgical method adopted this time: aPPV: anterior pars plana vitrectomy; GSL: goniosynechialysis ; PI: peripheral iridectomy; P: phacoemulsification cataract excision; I: Intraocular lens implantation

### Anterior chamber, vision, intraocular pressure, and eye medication

As shown in Figs. 1, 2, 3, 4 and 5, stable anterior chamber was formed in the five operated eyes (central anterior chamber depth > 4CT and peripheral anterior chamber depth > 1/2 CT).

In terms of vision improvement, only one eye showed an improvement to 0.2, while the other four did not demonstrate significant changes compared to pre-surgery levels. The preoperative examination of the fundus could not provide detailed information due to unclear refractive media. However, the postoperative fundus examination revealed pale optic discs in four eyes, indicating potential compression of the optic nerve due to elevated intraocular pressure caused by malignant glaucoma. Only one eye experienced fluctuating IOP at approximately 30 mmHg, but the patient did not report any discomforts such as swelling or pain. Four patients continued to receive cycloplegia treatment after surgery, ranging from once a day to once every other day. Three eyes still required the administration of local IOP-lowering drops following the surgical procedure (Table 1).

### Post-surgery complications

All patients underwent successful surgeries, without encountering any significant complications. However, one eye necessitated an additional procedure called transscleral cyclophotocoagulation (TCP) due to poor control of its IOP post-surgery. The IOP in this particular eye exceeded 40 mmHg and caused eye pain. Following the TCP treatment, the IOP in that eye was subsequently managed at approximately 30 mmHg and the patient did not report any discomforts such as swelling or pain.

## Discussion

Malignant glaucoma, first described by Von-Graefe in 1869 [5], continues to be a topic of ongoing research, as its pathogenesis remains incompletely understood despite more than a century of investigation. The modern understanding is as follows: When the drainage of aqueous fluid is obstructed for any reason, it can be forced to flow in the wrong direction, either into or behind the vitreous cavity. This generates a difference in pressure between the anterior chamber and the posterior chamber, leading to the forward movement of the lens-iris diaphragm and a shallowing of the anterior chamber. Subsequently, angle closure occurs, resulting in an elevated IOP [1, 2, 6]. The misdirection of aqueous fluid has a complex pathogenesis, believed to be closely associated with abnormal anatomical relations between the ciliary body, lens, and anterior hyaloid membrane. Currently, most experts consider malignant glaucoma to encompass any factor (such as inflammation, miotic use, and surgery) that causes the forward movement of the lens-iris diaphragm and blockages between different structures. These blockages can occur between the ciliary body and lens (classic malignant glaucoma), between the iris and lens capsular membrane, between the iris and anterior hyaloid membrane (without lens eye), or between the ciliary body and anterior hyaloid membrane. These blockages prevent the posterior chamber’s aqueous fluid from being discharged forward; resulting in its forced backflow into the vitreous cavity, where it accumulates and forms a water capsule. This further pushes the lens iris diaphragm forward, leading to a shallower anterior chamber and increased IOP. The blockage can be functional or organic. In early stages of malignant glaucoma, the blockage is reversible since there are no synechiae and other organic changes, and most cases can be relieved with cycloplegia treatment. However, in secondary malignant glaucoma, organic blockage always occurs, involving not only pathological changes such as vitreous body swelling and forward movement of the lens iris diaphragm but also the inflammatory process. This leads to irreversible blockage due to synechiae between the iris and lens, as well as between the ciliary body and the lens or anterior hyaloid membrane. Surgical treatment is typically necessary to alleviate the condition [7, 8].

In cases of early malignant glaucoma, drug treatment options can be explored. Along with the use of local steroidal anti-inflammatory drugs, cycloplegia, aqueous suppressant medication, and hypertonic dehydration drugs can be administered [9–11]. If the condition does not show improvement within 3 to 5 days of drug treatment initiation, laser or surgical interventions may be considered. Laser treatment typically involves the use of Nd:YAG (neodymium-doped yttrium aluminum garnet) laser to dissect the lens capsular membrane and anterior hyaloid membrane, thereby restoring the flow of aqueous fluid between the anterior and posterior segments [12–14]. Surgical approaches often include vitrectomy and phacoemulsification cataract excision, aiming to unblock both the anterior and posterior segments [15–19].

While timely treatment for malignant glaucoma can effectively control IOP and deepen the anterior chamber in most patients, there are cases where treatment is delayed for various reasons, resulting in prolonged absence of the anterior chamber. The prolonged absence of the anterior chamber is commonly observed in patients after glaucoma filtering surgery, as well as those with old uveitis, pupillary block with or without lens, neo-vascular glaucoma, and ocular trauma. Failure to provide timely treatment for shallow or absent anterior chambers in the early stages of these diseases, due to insufficient clinical experience or other subjective factors, can lead to the long-term absence of the anterior chamber. This condition not only contributes to the development of complicated cataracts and secondary glaucoma but can also result in permanent vision loss in severe cases. It is well known that the absence of the anterior chamber can lead to angle closure, impairing trabecular function, and cause corneal endothelial injury, potentially resulting in keratitis bullosa. Furthermore, the flow of aqueous liquid into the vitreous cavity further exacerbates malignant glaucoma by failing to reach the anterior chamber. In patients who have undergone glaucoma surgery, prolonged absence of the anterior chamber can lead to scarring of the filtering channel, loss of filtering function, uncontrolled IOP, metabolic disturbances in the lens, and complicated cataracts. Some patients may undergo multiple ciliary photocoagulation (TCP), develop scleral staphyloma, experience eyeball atrophy, and eventually require enucleation of the affected eyeball. Currently, there is no literature available to guide the use of active surgical treatment for such patients who are unlikely to recover their vision.

The misdirection of aqueous fluid in malignant glaucoma causes the absence of anterior chamber, resulting in elevated IOP. Interestingly, in this study, two cases who did not receive anti-glaucoma drops had normal baseline IOP prior to surgery. It is possible for some patients with malignant glaucoma to experience a superficial detachment of the ciliary body, which cannot be detected by UBM or B-scan imaging. Consequently, the IOP is reduced, and certain patients with malignant glaucoma may exhibit normal IOP levels.

The existing literature has consistently reported that patients with malignant glaucoma showed improved vision and IOP after surgical treatment [20, 21]. Without intervention, these patients would likely have atrophy of the eyeball due to the prolonged absence of the anterior chamber, along with dis-comfort in the affected eye. However, the long-term absence of the anterior chamber can lead to severe inflammatory reactions of the anterior segment, resulting in complications such as synechiae, iris neovascularization, and decompensation of the corneal endothelium. This increases the complexity of surgery and makes it challenging to predict the post-surgical recovery. Therefore, the question arises whether these patients should undergo surgical treatment. In this research, all the patients achieved anatomical replacement of anterior segment tissues through surgery but showed insignificant improvement in vision, except for the affected eye of Case 4. However, even though Case 4 developed a shallow anterior chamber up to 8 months after anti-glaucoma surgery, she complained of repeated drops in vision. Therefore, it is uncertain when the complete disappearance of the anterior chamber occurred. The long-term absence of the anterior chamber could result in corneal endothelium injury. However, in this research, all patients did not exhibit corneal endothelium decompensation or experience subjective eye discomfort after surgery. Therefore, if the patient is young, can afford the treatment, expresses a strong personal wish for treatment, and the medical institution has the necessary technical capabilities, surgical treatment should be considered, even if the chances of vision improvement are low. If elevated IOP persists after surgery, it can be lowered using cyclophotocoagulation. Future research will include extended follow-up periods to observe any changes. For patients with a prolonged absence of the anterior chamber, the research will be more cautious in adopting surgical methods, and detailed communication with the patients will be conducted prior to surgery.

## Conclusion

Malignant glaucoma with a prolonged absence of the anterior chamber is relatively rare in clinical practice. However, it poses significant risks as a long-term high IOP without the anterior chamber can cause irreversible damage to the eye’s structure and function. This not only leads to vision loss but is also accompanied by eye redness and pain, photophobia, a series of complaints of discomfort, and some abnormalities in appearance, which significantly impact the patient’s quality of life.

The present study focuses on the surgical treatment of patients with malignant glaucoma with the long-term absence of the anterior chamber. Postoperative examinations have shown that the anterior chamber remains stable, and the patients do not experience ocular discomfort. However, there is limited improvement in visual acuity. Even after surgery, the use of cycloplegic agents is still necessary to maintain the stability of the anterior chamber, and local IOP-lowering eye drops are required to help control the IOP. Through anatomical restoration and effective IOP control, patients experienced a reduction in complaints related to discomfort and appearance abnormalities. Therefore, surgical intervention can be considered for patients who do not respond well to conservative treatments if they have a strong desire for surgery and the medical facility has the necessary technical expertise.

## Data Availability

The data used to support the findings of this study are available from the corresponding author upon request.

## References

[1] Thompson AC, Vu DM, Postel EA, Challa P. Factors impacting outcomes and the time to recovery from malignant Glaucoma. Am J Ophthalmol. 2020 Jan;209:141–50. 10.1016/j.ajo.2019.07.023. Epub 2019 Aug 1. PMID: 31377283. 10.1016/j.ajo.2019.07.023 31377283

[2] Foreman-Larkin J, Netland PA, Salim S (2015). Clinical management of malignant Glaucoma. J Ophthalmol.

[3] Lois N, Wong D, Groenewald C. New surgical approach in the management of pseudophakic malignant glaucoma. Ophthalmology. 2001 Apr;108:780–3. 10.1016/s0161-6420(00)00642-4. Epub 2000 Nov 28. PMID: 11297497. 10.1016/s0161-6420(00)00642-4 11297497

[4] Madgula IM, Anand N. Long-term follow-up of zonulohyaloido-vitrectomy for pseudophakic malignant glaucoma. Indian J Ophthalmol. 2014 Dec;62:1115–1120. doi: 10.4103/0301-4738.149128. Epub: 2014 June 11. PMID:25579353. 10.4103/0301-4738.149128 PMC4313489 25579353

[5] von Graefe A. Beitrage zur pathologie und therapie des glaucoms. Arch fur Ophthalmol 1869;15:108–252.

[6] Kaplowitz K, Yung E, Flynn R, Tsai JC. Current concepts in the treatment of vitreous block, also known as aqueous misdirection. Surv Ophthalmol. 2015 May-Jun;60(3):229–41. 10.1016/j.survophthal.2014.12.004. Epub 2014 Dec 27. PMID: 25639795. 10.1016/j.survophthal.2014.12.004 25639795

[7] Levene RZ. Current concepts of malignant glaucoma. Ophthalmic Surg. 1986 Aug;17(8):515–8. 520. PMID: 3528972. 3528972

[8] Muqit MM, Menage MJ. Malignant glaucoma after phacoemulsification: treatment with diode laser cyclophotocoagulation. J Cataract Refract Surg. 2007 Jan;33(1):130-2. doi: 10.1016/j.jcrs.2006.07.041. PMID: 17189808. 10.1016/j.jcrs.2006.07.041 17189808

[9] Simmons RJ, Br JO. 1972 Mar;56(3):263–72. doi: 10.1136/bjo.56.3.263. PMID: 5032764; PMCID: PMC1208763. 10.1136/bjo.56.3.263 PMC1208763 5032764

[10] Ruben S, Tsai J, Hitchings RA. Malignant glaucoma and its management. Br J Ophthalmol. 1997 Feb;81(2):163–7. 10.1136/bjo.81.2.163. PMID: 9059253; PMCID: PMC1722113. 10.1136/bjo.81.2.163 PMC1722113 9059253

[11] Dave P, Senthil S, Rao HL, Garudadri CS. Treatment outcomes in malignant glaucoma. Ophthalmology. 2013 May;120(5):984–90. 10.1016/j.ophtha.2012.10.024. Epub 2013 Jan 31. PMID: 23375590. 10.1016/j.ophtha.2012.10.024 23375590

[12] Little BC, Hitchings RA. Pseudophakic malignant glaucoma: Nd:YAG capsulotomy as a primary treatment. Eye (Lond). 1993;7 (Pt 1):102-4. doi: 10.1038/eye.1993.21. PMID: 8325397. 10.1038/eye.1993.21 8325397

[13] Brown RH, Lynch MG, Tearse JE, Nunn RD. Neodymium-YAG vitreous surgery for phakic and pseudophakic malignant glaucoma. Arch Ophthalmol. 1986 Oct;104(10):1464-6. doi: 10.1001/archopht.1986.01050220058026. PMID: 3767675. 10.1001/archopht.1986.01050220058026 3767675

[14] Tsai JC, Barton KA, Miller MH, Khaw PT, Hitchings RA (1997). Surgical results in malignant glaucoma refractory to medical or laser therapy. Eye (Lond).

[15] Yu X, Zhao Z, Zhang D, Yang X, Sun N, Lin Y, Zhang J, Fan Z. Anterior vitrectomy, phacoemulsification cataract extraction and irido-zonulo-hyaloid-vitrectomy in protracted acute angle closure crisis. Int Ophthalmol. 2021 Sep;41(9):3087–97. 10.1007/s10792-021-01874-2. Epub 2021 Apr 27. PMID: 33905050; PMCID: PMC8076881. 10.1007/s10792-021-01874-2 PMC8076881 33905050

[16] Pathak Ray V, Gulati I, Choudhari N. Intra-operative Ostial Irido-Zonulo-Hyaloido-Vitrectomy with primary posterior capsulectomy for Prevention of Post-Operative Aqueous Misdirection in Combined Phaco-Trabeculectomy in Primary Angle Closure Glaucoma. Curr Eye Res. 2019 Oct;44(10):1087–90. Epub 2019 Jun 14. PMID: 31136195. 10.1080/02713683.2019.1625409 31136195

[17] Byrnes GA, Leen MM, Wong TP, Benson WE. Vitrectomy for ciliary block (malignant) glaucoma. Ophthalmology. 1995 Sep;102(9):1308-11. doi: 10.1016/s0161-6420(95)30870-6. PMID: 9097767. 10.1016/s0161-6420(95)30870-6 9097767

[18] Sharma A, Sii F, Shah P, Kirkby GR. Vitrectomy-phacoemulsification-vitrectomy for the management of aqueous misdirection syndromes in phakic eyes. Ophthalmology. 2006 Nov;113(11):1968-73. doi: 10.1016/j.ophtha.2006.04.031. PMID: 17074562. 10.1016/j.ophtha.2006.04.031 17074562

[19] Liu X, Li M, Cheng B, Mao Z, Zhong Y, Wang D, Cao D, Yu F, Congdon NG. Phacoemulsification combined with posterior capsulorhexis and anterior vitrectomy in the management of malignant glaucoma in phakic eyes. Acta Ophthalmol. 2013 Nov;91(7):660-5. doi: 10.1111/j.1755-3768.2012.02451.x. Epub 2012 Jun 7. PMID: 22676180. 10.1111/j.1755-3768.2012.02451.x 22676180

[20] Rękas M, Krix-Jachym K, Żarnowski T (2015). Evaluation of the effectiveness of Surgical treatment of malignant Glaucoma in pseudophakic eyes through partial PPV with establishment of communication between the Anterior Chamber and the vitreous cavity. J Ophthalmol.

[21] Wu ZH, Wang YH, Liu Y. Management strategies in malignant glaucoma secondary to antiglaucoma surgery. Int J Ophthalmol. 2016 Jan 18;9(1):63 – 8. doi: 10.18240/ijo.2016.01.11. PMID: 26949612; PMCID: PMC4768516. 10.18240/ijo.2016.01.11 PMC4768516 26949612

